# Obstructive Sleep Apnea Syndrome (OSAS), Metabolic Syndrome and Mental Health in Small Enterprise Workers. Feasibility of an Action for Health

**DOI:** 10.1371/journal.pone.0097188

**Published:** 2014-05-08

**Authors:** Sergio Garbarino, Nicola Magnavita

**Affiliations:** 1 Department of Neuroscience, Rehabilitation, Ophthalmology, Genetics and Maternal-Infantile Sciences (DINOGMI), and Department of Health Sciences, University of Genoa, Genoa, Italy; 2 Department of Public Health, Section of Occupational and Environmental Medicine, Università Cattolica del Sacro Cuore, Roma, Italy; Shanghai Mental Health Center, Shanghai Jiao Tong University School of Medicine, China

## Abstract

**Objective:**

To determine the frequency of obstructive sleep apnea syndrome (OSAS), metabolic syndrome and common mental disorders in the working population of 11 small enterprises and the feasibility of a program of action for health.

**Method:**

The clinical risk of OSAS, the prevalence of metabolic syndrome, and the level of psychological disorders were assessed during routine medical examination at the workplace in 2012. The response to medical advice was assessed in 2013.

**Results:**

12.3% of the workers were suspected of being affected by OSAS. One or more components of metabolic syndrome were present in 24.5% of cases. OSAS in “healthy” workers was significantly associated with the presence of one or more components of metabolic syndrome (OR = 3.83; 95%CI 1.45–10.13) and with a psychological disorders score in the highest quartile (OR = 4.67; 95%CI = 1.72–12.64). Workers with suspected OSAS were reluctant to follow advice about undergoing further tests under the NHS. However, in some cases, confirmation of the OSAS diagnosis and subsequent treatment led to an improvement in metabolic condition.

**Conclusion:**

Although participation in treatment was limited, anecdotal cases support the idea that prevention of obstructive sleep apnea in the workplace might be useful for workers’ health.

## Introduction

Obstructive sleep apnea syndrome (OSAS) is a common disorder caused by repeated episodes of airflow cessation (apneas) leading to arterial hypoxemia and sleep disruption [1]. This syndrome may have a number of consequences that deeply affect the quality of life, such as excessive daytime sleepiness, neurocognitive deterioration, and endocrinologic and metabolic effects [2]. Metabolic syndrome (MS) that is, itself, emerging as a highly prevalent public health problem, may be linked with OSAS. In fact, the presence of OSAS may increase the risk of developing some aspects of metabolic syndrome [3], [4]. Moreover, clinical studies have shown a statistically significant correlation between the severity of OSAS and obesity, hypertension, diabetes mellitus, dyslipidaemia and metabolic syndrome [5]. However, little is known about the possible association between early-stage OSAS and MS components in active workers.

Only limited knowledge is also available concerning the relationship between the presence of OSAS and psychological problems, although this syndrome is known to have an impact on neurocognitive functioning. Employees with OSAS run a major risk of long-term work disability caused by injuries and mental disorders [6]. Self-reported symptoms of OSAS are an independent risk factor for subsequent long-term sick leave and permanent work disability [7]. Both OSAS and hypertension related to this syndrome are significant predictors of impairment in workers [8]. However, all the above studies have focused on advanced cases of OSAS. Again, nothing is known about the mental health of active workers with previously undiagnosed OSAS.

The aim of occupational medicine is to continuously improve the health of workers. For this reason, many health promotion campaigns are conducted in major companies. The main objective of these programs is to identify a risk factor that can be removed or minimized, thereby improving occupational health. OSAS may constitute a risk factor that requires an occupational prevention program. The first objective of this study was therefore to evaluate the importance of OSAS as a risk factor. In order to do this we aim to assess the prevalence of OSAS among previously undiagnosed “healthy” subjects working in various fields and study the association between OSAS and MS components and mental health.

Before undertaking a campaign to promote health, it is necessary to check the availability of resources in the country and productive area where you intend to introduce measures of prevention. In Italy, industrial production is characterized mainly by the presence of small businesses with fewer than 50 workers. Health promotion in these companies is more difficult than in large firms, owing to the lack of financial resources and organizational structures needed to support it.

The second objective of our study was to ascertain whether a health promotion program, conducted by a clinician performing health surveillance without the use of additional resources can achieve measurable results in a sample of small companies in the Latium region of Italy.

## Methods

### Population

The baseline study was conducted in the first 4 months of 2012 on workers called for a routine medical examination in the workplace. The data in this study are partially drawn from the multicenter project SOLARIS (OSAS Screening of Workers in High Risk of Injury or Accident), coordinated by the University of Genoa. The workers belonged to 11 small companies, each employing between 10 and 51 workers. The productive sectors involved were: insurance, fuel distribution, home care, education, laboratory, wholesale, supermarkets, electronics and hotels. Subjects with a previous diagnosis of OSAS or respiratory disorders were excluded from the investigation. 204 out of 209 eligible subjects (97.6%) participated in the survey.

In 2013, 198 of these 204 workers (97.1%) were still working in the same company and were again called to medical examination in the workplace. During the visit, which is compulsory, workers who had been advised to do investigations for OSAS were asked the outcome. None of the subjects with suspected OSAS was lost at follow-up. The overall participation rate at follow-up was 94.7%.

### Questionnaires

Within the project, SOLARIS workers were asked to complete two short questionnaires: the sleep disorder score (SDS) [9], and the Epworth Sleepiness Scale (ESS) [10].

The SDS test, used to aid diagnosis of OSAS, contains 4 items that respectively refer to nocturnal snoring, apnea, nocturnal awakenings, and dry mouth in the morning. A global score was evaluated for each subject by averaging the numeric values associated with all 4 items, and an SDS score of over 2 was considered to be pathological.

The ESS test is a simple instrument for evaluating excessive daytime sleepiness. It is an eight-item questionnaire which provides a simple and inexpensive measurement of tendency to sleep in different daily life situations. For each situation, subjects could express a range of numeric values, from 0 to 3, thus obtaining a total score ranging from 0 (no daytime sleepiness) to 24 (the highest level of daytime sleepiness). Normally, ESS scores of over 10 were considered to indicate excessive daytime sleepiness [11]. However, studies performed with the Italian version of the questionnaire showed that 12 and 17 were the ESS cut-off scores with the best sensitivity and specificity [12].

Psychological distress was measured by the Italian version [13] of the General Health Questionnaire [14]. This test is made up of 12-items. Each item is accompanied by four possible responses which can be scored on a Likert scale from 1 to 4. The total score can yield a continuous variable ranging from 12 to 48. In the present study, GHQ12 had a Cronbach alpha = 0.849. For the purposes of statistical analysis, the values obtained with the GHQ12 were divided into quartiles and the highest quartile was taken as an indicator of psychological problems. The population was thus divided into two groups: those with psychological problems (GHQ in the highest quartile, i.e. >24) and all the others.

In addition to gender and age, each subject declared smoking status (current smoker/non-smoker), alcohol use (divided into four classes: 1 = I do not drink alcohol; 2 = I drink up to seven units of alcohol per week, i.e. one in the daytime; 3 = I drink from eight to sixteen units of alcohol per week, i.e. two per day; 4 = I drink more than sixteen units per week, i.e. more than 2 per day); physical exercise (at least 30 minutes of vigorous physical activity, 1 = at least 3 times a week; 2 = twice a week; 3 = once a week; 4 = never).

During medical examinations, body mass index (BMI; kg/m^2^), neck circumference (NC; cm), thyromental, or neck-chin angle (N-CA; grades) [15], and visibility of pharyngeal structures according to the Mallampati classification [16] were measured. The Mallampati classification is a rough estimate of tongue size related to the oral cavity. This was initially proposed as a simple, reproducible, and reliable preanesthetic airway assessment method. Besides being a sign of difficult tracheal intubation, Mallampati class 3 or 4 is an independent predictor for the presence of obstructive sleep apnea [17].

OSAS was suspected when there were at least 2 positive responses to the sleep disorders scale (SDS) associated with at least 1 above the anthropomorphic threshold measured among the following parameters: BMI (>30 kg/m^2^); NC (>43 cm in male and >41 cm in female workers); N-CA (>110°) and Mallampati (3 or 4).

After collecting the Solaris project data, subjects were further examined to evaluate the presence of metabolic syndrome. According to the International Diabetes Federation (IDF) guide [18], metabolic syndrome is identified by the following criteria: central obesity (defined as BMI>30 kg/m^2^, or increased waist circumference with ethnicity-specific values); elevated triglyceride level: >150 mg/dL (1.7 mmol/L), or specific treatment for this lipid abnormality; reduced HDL cholesterol: <40 mg/dL (1.03 mmol/L) in males, <50 mg/dL (1.29 mmol/L) in females, or specific treatment for this lipid abnormality; high blood pressure (BP): systolic BP>130 or diastolic BP>85 mm Hg, or treatment of previously diagnosed hypertension; high fasting plasma glucose (FPG): >100 mg/dL (5.6 mmol/L), or previously diagnosed type 2 diabetes.

Workers with suspected OSAS were invited to undergo second level studies (specialist neurological or pulmonary examination and possibly, depending on the advice of the specialist, imaging studies such as polysomnography (PSG). Adherence to this invitation was verified a year later during routine medical examination in the workplace.

The article metadata of this study are deposited in Dryad.

### Ethics Statement

Workers signed written consent. The Ethics Committee of the Università Cattolica del Sacro Cuore of Rome approved the study design and the consent form.

### Statistics

Analyses were performed using IBM/SPSS for Windows (rel. 20.0). First of all, the data collected were analyzed using common statistics. The results obtained for the two genders were compared using the Student’s t test for independent data (comparison of means) or the chi-square test (comparison of proportions). Logistic regression analysis was used to study the association between suspected OSAS and the presence of at least one metabolic syndrome components (obesity, hypertension, dyslipidemia, diabetes). The association between suspected OSAS and the presence of psychological problems (GHQ12 score in the highest quartile) was also studied using logistic regression analysis. In both the aforementioned analyses, personal factors (age, sex, smoking, alcohol use, physical exercise) were entered as confounding factors.

## Results

The clinical and demographic characteristics of the subjects, both as a group and classified by gender, are reported in Table 1. The descriptive statistics indicate a group of experienced workers (13.8±9.1 years) with a low number (37.3%) of smokers and a normal mean BMI range (24.5±4.3 Kg/cm^2^). 4.3% (n = 7) of subjects had an ESS score >10, indicating daytime sleepiness.

**Table 1:** Medical and demographic data for participants.

Variable	Male	Female	*p*	Whole group
Gender, N (%)	108 (52.9)	96 (47.1)		204 (100)
Age, years (mean+s.d.)	41.3+9.4	40.8+9.0	n.s.^1^	41.1+9.2
Length of work, years	13.2+9.7	14.6+8.3	n.s.^1^	13.8+9.1
Smoker, N (%)	46 (42.6)	30 (31.2)	n.s.^2^	76 (37.3)
Alcohol, 1–7 units/week	51 (47.7)	28 (29.2)	<0.011^2^	79 (38.9)
Alcohol, 8–16 units/week	7 (6.5)	4 (4.2)		11 (5.4)
BMI (mean+s.d.)	25.8+3.7	23.0+4.5	<0.000^1^	24.5+4.3
Enlarged neck circumference	21 (19.4)	2 (2.1)	<0.000^2^	23 (11.3)
Increased neck-chin angle	44 (40.7)	22 (22.9)	<0.007^2^	66 (32.4)
Mallampati grade 3 or 4	24 (22.2)	10 (10.4)	<0.024^2^	34 (16.7)
ESS (range 0–24)	3.5+2.5	3.9+3.3	n.s.^1^	3.7+2.9
SDS (range 0–4)	0.99+1.0	0.69+0.85	<0.025^1^	0.85+0.97
GHQ (range 12–48)	21.9+3.5	22.7+5.6	n.s.^1^	22.2+4.6

1 = Student’s t test, 2 = chi square.

In the gender comparison, males reported a significantly higher consumption of alcohol than females (p<0.011). A greater frequency of enlarged neck circumference, increased neck-chin angle, and Mallampati grade 3–4 (p<0.024) was found in males than in females. The score of the SDS questionnaire on sleep disorders was significantly higher in males than in females (p<0.025), whereas no significant difference between genders was observed in the scores of the ESS questionnaire on sleepiness and the GHQ on mental health.

We identified 25 subjects (12.3%) with a clinical suspicion of OSAS. None of the workers reached the level of 17 points on the ESS scale, considered to be the cut-off point for greater diagnostic specificity. Only 3 workers with suspected OSAS were affected by mild sleepiness (ESS score >10). Two of the latter had parameters indicating a complete metabolic syndrome (obesity, plus two other metabolic disorders).

In data regarding MS only, 50 subjects (24.5%) presented one or more risk factors (obesity, dyslipidemia, hypertension, or diabetes). The prevalence of workers with suspect OSAS having at least one MS components was 52.0%. Logistic regression analysis showed that clinical suspicion of OSAS was significantly associated with the presence of metabolic disorders (OR = 3.83; 95%CI 1.45–10.13) in a model that included other personal factors (sex, age, smoking habit, alcohol consumption, and sedentary life). Age, male gender and lack of physical activity were significantly associated with the presence of metabolic syndrome components (Table 2).

**Table 2:** Association between suspected OSAS and personal factors (age, sex, smoking habit, alcohol use, lack of physical exercise) and the presence of at least one components of metabolic syndrome (obesity, hypertension, dyslipidemia, diabetes).

	Predictors			*p* value
	OR	95%CI		
OSAS	3.831	1.449–10.129		0.007
Age	1.073	1.031–1.116		0.001
Sex	0.395	0.185–0.842		0.016
Smoker	1.349	0.645–2.822		0.426
Alcohol use	0.777	0.414–1.458		0.432
Lack of physical activity	1.878	1.278–2.761		0.001

Psychological problems, as measured by the GHQ12 mean score, were significantly higher in OSAS than in non-OSAS subjects (25.7+7.2 vs. 21.8+4.0, test t p<0.000). Workers with OSAS had a significantly increased risk of having a GHQ12 score in the highest quartile, (OR = 4.67; 95%CI = 1.72–12.64) in a multivariate logistic regression model that included age, gender, smoking habit, alcohol use, and physical activity (Table 3).

**Table 3:** Associations between suspected OSAS and personal factors (age, sex, smoking habit, alcohol use, lack of physical exercise) and GHQ12 score in the highest quartile.

	Predictors		*p* value
	OR	95%CI	
OSAS	4.668	1.724–12.640	0.002
Age	1.015	0.975–1.057	0.463
Sex	3.656	1.619–8.257	0.002
Smoker	1.306	0.604–2.826	0.497
Alcohol use	1.236	0.667–2.290	0.500
Lack of physical activity	0.973	0.697–1.357	0.870

During the follow-up survey carried out in 2013, all workers who had been sent to their own doctors with a diagnosis of suspected OSAS, and an invitation to perform additional tests under the NHS, were questioned on the outcome. A worker with severe systolic-diastolic hypertension resistant to therapy had been diagnosed with OSAS. Effective treatment of OSAS with continuous positive airway pressure for 3 months had significantly reduced blood pressure. Two workers with abdominal obesity had received confirmation of the OSAS diagnosis and were following positive airway pressure therapy associated with nutritional therapy. One worker had the OSAS diagnosis confirmed and had been treated with mandibular advance device therapy. In the case of another worker who had undergone further medical investigations, the diagnosis of OSAS had been ruled out. The majority of workers (20/25 = 80%) had not heeded the invitation of the occupational physician and had not carried out any further investigation. The reasons most frequently given for failing to adhere to the invitation were lack of time, lack of health service availability, and especially the conviction that they were in a satisfactory state of health.

## Discussion

The present study confirms that obstructive sleep apnea is a rather frequent and underdiagnosed condition, and that daytime sleepiness is not uncommon. This is very worrisome. Reduction in vigilance and attention could be fatal in some job categories such as drivers and operators of dangerous machines. Moreover, the risk of cardiovascular disease and the psychological problems associated with OSAS could lead to serious health consequences.

Although this is a small-scale study, our assumptions are also valid for selected populations. As we know, the occupational physician can only examine healthy workers. In fact, medical examination in the workplace is not possible during illness, even when it is of short duration. This can cause an undervaluation of the workers’ state of illness. Moreover, workers are selected from the general population. In fact, the so-called “healthy worker effect” refers precisely to the fact that the occupational population have a better level of health than the general population. Although we tested a selected population, we found a 12.3% prevalence of suspected OSAS, and 4.3% of workers reported feeling very drowsy during work.

In this sample, 24.5% suffered from obesity, or high blood pressure, diabetes or dyslipidemia. OSAS was significantly associated with these metabolic disorders. The cross-sectional nature of these observations prevents us from making causal considerations, however a recent review [19] shows that obesity predisposes to OSAS, and the increasing prevalence of OSAS is influenced by the worldwide ongoing epidemic of obesity [20]. It has been observed, indeed, that obesity and OSAS tend to cluster in the same workplace [21]. Many markers of cardiovascular risk, such as sympathetic activation, systemic inflammation, and endothelial dysfunction, are significantly increased in obese patients with OSAS compared to those without OSAS [22]–[25]. This fact suggests that OSAS is not simply an epiphenomenon of obesity. Moreover, findings from animal models and patients with OSAS show that intermittent hypoxia exacerbates the metabolic dysfunction of obesity, augmenting insulin resistance and nonalcoholic fatty liver disease [26], [27]. In patients with the metabolic syndrome, OSAS is independently associated with increased glucose and triglyceride levels as well as markers of inflammation, arterial stiffness, and atherosclerosis [28], [29]. Several cohort studies have consistently shown that OSAS is associated with increased cardiovascular mortality, independent of obesity [30]–[32]. Taken together, these results support the concept that OSA exacerbates the cardiometabolic risk attributed to obesity and metabolic syndrome. However, it has been demonstrated that recognition and treatment of OSAS may decrease the cardiovascular risk in patients with metabolic syndrome [33]–[36].

An interesting result of our study was the association observed between suspected OSAS and psychological problems. To our knowledge, this aspect has never been studied in previous research on patients with suspected OSAS although this syndrome is known to be linked to a number of complications such as psychiatric conditions that significantly impair the quality of life. For example, OSAS may be associated with depression in adolescents and with Down Syndrome in young adults [37]. These macroscopic effects are the result of deep physiological alterations. In fact, at cellular level, OSAS can cause intermittent hypoxia, hormonal imbalance, and/or systemic inflammation which may influence cognitive functions [38]. However, in patients with OSAS, mental fatigue and cognitive impairment are directly correlated to the severity of nocturnal disordered breathing [39]. Clearly, the severity of the pathology is very important because a cognitive consequence is expected only in severe and advanced cases, not in recently diagnosed OSAS patients with minimal co-morbidities [40], or in suspected cases identified during screening.

Mood disorders in patients with OSAS have also been studied, especially in relation to the way in which these problems may affect adherence to treatment; however the nature of the relationship between OSAS and depression and anxiety is still unclear [41]. In fact, recent research on depression and anxiety in obstructive sleep apnea syndrome led to inconsistent findings: prevalence figures fluctuated considerably for both depression (7–63%) and anxiety (11–70%) [42]. Moreover, the reported associations were often contrasting. In some cross-sectional studies, OSAS patients had higher scores for depression and anxiety than the control group [43], while in other studies, OSAS was not associated with severe symptoms of depression and anxiety [44]. These differences are probably due to the methods used to measure psychological disorders.

In the present study, we used a particularly sensitive tool to measure mental health. The GHQ is possibly the most common assessment of mental well-being [45]. In fact, it is used to detect minor psychiatric (non-psychotic) disorders in the general population and within community or non-psychiatric clinical settings. It focuses on two main areas: inability to carry out normal functions and the appearance of new and distressing psychological phenomena. The GHQ assesses the respondent’s current state and determines whether it differs from his or her usual state. Therefore, it is sensitive to short-term psychiatric disorders, but not to long-standing attributes of the respondent. This instrument, which was designed to detect psychiatric morbidity, has also performed very well in trans-cultural comparisons of community-based populations [46]. We can therefore assume that the GHQ measures an early, and possibly transient worsening of psychological conditions in workers with recent onset of sleep apnea. We conclude, in accordance with the literature, that the relationships between early-stage OSA and psychological disturbances are weak but significant.

In our study, prevalence values for metabolic syndrome were lower than those estimated in the general Italian population and other industrialized countries. The third National Health and Nutrition Examination Survey (NHANES III) recorded a 21.8% prevalence of metabolic syndrome in the total population [47]. In NHANES III the prevalence of abdominal obesity was approximately 50% in females and 30% in males. This was also higher than the value found in our study, as were the US population levels of impaired HDL-cholesterol, hyperglycemia and hypertension. The few data available for Italy confirm that metabolic syndrome and its components occur more frequently than was observed in our sample. The Italian Longitudinal Study on Aging (ILSA) reported a metabolic syndrome prevalence of 25.9% [48]. A study conducted in the Marche Region of Italy found a metabolic syndrome prevalence of 11.5% in the 36 to 42 age group and of 22.5% in subjects between the age of 43 and 60 years [49]. This demonstrated that in our sample group at least, the health level of employees was higher than that of the general population – a fact that must be taken into account in the planning of action for health. It also indicated that the subjects taking part in our study were not prompted in because they were suffering from one of the pathologies under investigation. This was important since we know that being affected by any morbid condition induces individuals to be more precise when recalling all the possible causal factors for that illness. This phenomenon, known as “recall bias”, can give rise to fictitious associations. Compared with what may occur in case-control studies, the limited number of sick subjects in our survey reduced the impact of such an effect.

Unfortunately, in our study, the majority of workers with suspected OSAS failed to respond to advice suggesting they undergo other tests to confirm the diagnosis. In the few cases in which subjects followed the advice of the occupational physician, a significant clinical improvement was observed.

This experience demonstrated that health promotion during compulsory health surveillance is effective in only a limited number of workers. The main reason for ignoring the physician’s advice seemed to be unawareness on the part of the workers of the importance of the detrimental effects of OSAS. A multilevel effort that includes specific information about the health risks associated with OSAS and incentives for workers who participate in the programs proposed, might effectively help to safeguard workers’ health. Since small enterprises lack financial resources and training structures, these services must be provided by public or consortium facilities.

The main limitation of this study concerned sample selection, as all the companies were located in the same region and were under the health surveillance of the same doctor (NM). These conditions obviously prevented us from extending our findings to other situations. One of the strengths of the study was the high level of participation at baseline. Another strength was the inclusion of general workers in the study population. Previous studies on the prevalence and risk factors of occupational OSAS had mainly targeted at-risk workers, so that results were not applicable to a more general population of workers. A further strength was the broad set of clinical issues examined; these included an objective evaluation of the risk of OSAS, an assessment of sleepiness, blood tests and clinical examination to diagnose metabolic syndrome and screening for psychological disorders. All these tests were included during the routine medical surveillance of workers without a significant burden of time for the performance of medical examinations.

The data collected show that OSAS is an important risk factor that is widely prevalent in the general population of workers, and that this syndrome is strongly correlated with metabolic syndrome and mental disorders. It is therefore an ideal candidate for health intervention.

Health promotion programs conducted in small enterprises and based exclusively on services provided by the NHS, without a spending commitment for training and incentives on the part of employers, have a beneficial but limited impact on the health of workers. Only a few workers accepted the invitation of the occupational physician to undergo more specialized tests to confirm or refute the diagnosis of OSAS. However, excellent results were obtained for those who followed this advice. We are confident that a prevention program comprising educational, health and organizational activities could actually prevent OSAS and its physical and psychological consequences in the general population of workers.
